# Vasectomy and Prostate Cancer Risk in the European Prospective Investigation Into Cancer and Nutrition (EPIC)

**DOI:** 10.1200/JCO.2016.70.0062

**Published:** 2017-03-06

**Authors:** Karl Smith, Byrne, Jose Maria Castaño, Maria Dolores Chirlaque, Hans Lilja, Antonio Agudo, Eva Ardanaz, Miguel Rodríguez-Barranco, Heiner Boeing, Rudolf Kaaks, Kay-Tee Khaw, Nerea Larrañaga, Carmen Navarro, Anja Olsen, Kim Overvad, Aurora Perez-Cornago, Sabine Rohrmann, Maria José Sánchez, Anne Tjønneland, Konstantinos K. Tsilidis, Mattias Johansson, Elio Riboli, Timothy J. Key, Ruth C. Travis

**Affiliations:** Karl Smith Byrne, Hans Lilja, Aurora Perez-Cornago, Timothy J. Key, and Ruth C. Travis, University of Oxford, Oxford; Kay-Tee Khaw, School of Clinical Medicine, University of Cambridge, Cambridge, Konstantinos K. Tsilidis and Elio Riboli, Imperial College London, London, United Kingdom; Jose Maria Castaño, Maria Dolores Chirlaque, and Carmen Navarro, Murcia Regional Health Council; Maria Dolores Chirlaque, IMIB-Arrixaca, Murcia; Jose Maria Castaño, Maria Dolores Chirlaque, Eva Ardanaz, Miguel Rodríguez-Barranco, Nerea Larrañaga, Carmen Navarro, and Maria José Sánchez, Center for Biomedical Research Network for Epidemiology and Public Health, Madrid; Antonio Agudo, Catalan Institute of Oncology-IDIBELL, Barcelona, Spain; Eva Ardanaz, Navarra Institute for Health Research, Pamplona; Miguel Rodríguez-Barranco and Maria José Sánchez, Universidad de Granada, Granada; Nerea Larrañaga, Public Health Division of Gipuzkoa, Regional Government of the Basque Country, Donostia, Spain; Hans Lilja, Memorial Sloan Kettering Cancer Center, New York, NY; Lund University, Malmö, Sweden; Heiner Boeing, German Institute of Human Nutrition, Potsdam-Rehbrücke; Rudolf Kaaks, German Cancer Research Center, Heidelberg, Germany; Anja Olsen and Anne Tjønneland, Danish Cancer Society Research Center, Copenhagen; Kim Overvad, Aarhus University, Aarhus, Denmark; Sabine Rohrmann, University of Zurich, Zurich, Switzerland; Konstantinos K. Tsilidis, University of Ioannina School of Medicine, Ionnina, Greece, and Mattias Johansson, International Agency for Research on Cancer, Lyon, France.

## Abstract

**Purpose:**

Vasectomy is a commonly used form of male sterilization, and some studies have suggested that it may be associated with an increased risk of prostate cancer, including more aggressive forms of the disease. We investigated the prospective association of vasectomy with prostate cancer in a large European cohort, with a focus on high-grade and advanced-stage tumors, and death due to prostate cancer.

**Patients and Methods:**

A total of 84,753 men from the European Prospective Investigation into Cancer and Nutrition (EPIC), aged 35 to 79 years, provided information on vasectomy status (15% with vasectomy) at recruitment and were followed for incidence of prostate cancer and death. We estimated the association of vasectomy with prostate cancer risk overall, by tumor subtype, and for death due to prostate cancer, using multivariable-adjusted Cox proportional hazards models.

**Results:**

During an average follow-up of 15.4 years, 4,377 men were diagnosed with prostate cancer, including 641 who had undergone a vasectomy. Vasectomy was not associated with prostate cancer risk (hazard ratio [HR], 1.05; 95% CI, 0.96 to 1.15), and no evidence for heterogeneity in the association was observed by stage of disease or years since vasectomy. There was some evidence of heterogeneity by tumor grade (*P* = .02), with an increased risk for low-intermediate grade (HR, 1.14; 95% CI, 1.01 to 1.29) but not high-grade prostate cancer (HR, 0.83; 95% CI, 0.64 to 1.07). Vasectomy was not associated with death due to prostate cancer (HR, 0.88; 95% CI, 0.68 to 1.12).

**Conclusion:**

These findings from a large European prospective study show no elevated risk for overall, high-grade or advanced-stage prostate cancer, or death due to prostate cancer in men who have undergone a vasectomy compared with men who have not.

## INTRODUCTION

Vasectomy is a commonly used form of male sterilization that has been performed globally in an estimated 40 million to 60 million men.^1^ Although a meta-analysis of five prospective cohort studies^2^ and a subsequent analysis in the Cancer Prevention Study II (CPS-II)^3^ found no significant elevated risk of prostate cancer associated with vasectomy, a recent investigation in the Health Professionals Follow-Up Study (HPFS) has reported a significant increase in risk of high-grade (hazard ratio [HR], 1.22; 95% CI, 1.03 to 1.45) and advanced-stage prostate cancer (HR, 1.20; 95% CI, 1.03 to 1.40) associated with having a vasectomy.^4^

Various biologic mechanisms have been suggested to explain an association of vasectomy with prostate cancer, including immunologic effects,^5^ cellular proliferation,^6^ and sex hormone imbalances.^7^ However, none has been clearly supported in humans. Differences in health-seeking behaviors have been suggested as a possible explanation for any association^1,2,8,9^; men who have had a vasectomy may be more likely to monitor their health, have a prostate-specific antigen (PSA) test, and be diagnosed with prostate cancer.^4,10^

The current study investigated the association between vasectomy and prostate cancer risk in the European Prospective Investigation into Cancer and Nutrition (EPIC), with a focus on tumor stage and grade, and death due to prostate cancer. We also examined the cross-sectional associations of vasectomy with PSA testing and with circulating concentrations of seminal proteins.

## PATIENTS AND METHODS

### Study Population

EPIC included 142,239 men recruited at 19 centers in eight European countries (Denmark, Germany, Greece, Italy, The Netherlands, Spain, Sweden, and the United Kingdom).^11^ Recruitment was between 1992 and 2000, and at enrolment participants were mostly aged between 35 and 79 years. All participants provided written informed consent. Source populations were generally identified by geographic administrative boundaries constituting a sample of convenience invited in person or by mail to complete baseline EPIC questionnaires. Center-specific recruitment criteria are outlined by Riboli et al.^11^ There was modest heterogeneity of study practices by recruitment centers; estimates of response rates were between approximately 22% and 60% across centers.^12-15^ Approval for the study was obtained from the ethical review boards of the participating institutions and the International Agency for Research on Cancer.

Information about lifestyle factors (ie, smoking status, physical activity, and alcohol consumption), sociodemographic characteristics (ie, marital status and educational attainment), diet, and medical history (including vasectomy status and age at vasectomy) was collected via questionnaires at recruitment. For 6,771 (97.3%) of 6,961 men in the EPIC-Oxford cohort who completed a follow-up questionnaire, history of PSA testing and age at PSA test were collected 10 years after recruitment. Weight and height were measured at recruitment, except for part of the Oxford cohort for whom height and weight were self-reported. Body mass index (BMI) was calculated as weight in kilograms divided by height in meters squared.

This analysis includes 84,753 men from Denmark, Germany, Spain, and the United Kingdom who provided information on vasectomy status at recruitment; data on vasectomy were missing for 712 men for these countries. Information on vasectomy was not available for men in Italy, The Netherlands, or Sweden (n = 45,960). Additionally, in the Greek recruitment center, only four men had had a vasectomy and there were no exposed incident cases of prostate cancer; therefore, because all analyses were stratified by recruitment center, Greek participants were excluded (n = 10,814).

### Ascertainment of Prostate Cancer

Information on cancer diagnosis was obtained from national and regional registries for Denmark, Spain, and the United Kingdom. For Germany, active follow-up, including inquiries by mail or telephone to participants, municipal registries, regional health departments, physicians, and hospitals, was used. Information on death, including death due to prostate cancer as the underlying cause, was obtained from death certificates; available evidence suggests information on death due to prostate cancer is accurate.^16-19^ For analyses of incidence, follow-up continued from date of recruitment to date of any primary cancer diagnosis, death, or last completed follow-up (Denmark, December 31, 2012; Germany, January 5, 2011; Spain, October 19, 2013; and the United Kingdom, December 31, 2012), whichever was first. For analyses of death due to prostate cancer, follow-up continued until death or date of last completed follow-up. During the follow-up period, 4,377 men developed prostate cancer (International Classification of Diseases 10th revision codes, C61^20^).

Cancer-stage information was available for 2,733 (63.3%) of participants. The number of participants with tumor-node-metastasis (TNM) staging of T1-T3, N0/Nx, and M0/Mx, or stage coded in the recruitment center as having localized disease was 2,100; the number of participants identified as having advanced prostate cancer (T4 and/or N1-N3, and/or M1, or stage coded in the recruitment center as metastatic) was 633. Grade information was available for 2,986 (68.2%) of participants. The number of participants with low-intermediate grade (Gleason score < 8, or grade coded as well, moderately, or poorly differentiated) was 2,438. The number of participants identified as high-grade prostate cancer (Gleason score ≥ 8, or grade coded as undifferentiated) was 544. There was a small difference in the frequency of vasectomy between patients with prostate cancer who did and did not have tumor subtype information; thirteen percent of participants with information on tumor stage had had a vasectomy, compared with 17% of participants without tumor stage information; and 14% of participants with information on tumor grade had had a vasectomy, compared with 16% of participants without tumor grade information. By the end of follow-up, 15,285 men had died, of whom 632 had died of prostate cancer.

### Laboratory Assays

Assay data were available for men in the EPIC cohort who had been selected as controls in an unpublished, matched nested case-control study of prostate cancer. Each control had been selected at random from the cohort of men who were alive and free of cancer (excluding nonmelanoma skin cancer) at the time of diagnosis of their index case, using an incidence density sampling protocol (further details on matching methods can be found in Travis et al).^21^ Immunoassay measurements for total PSA, free PSA,^22^ intact PSA, human kallikrein 2 (hK2),^23,24^ and microseminoprotein-β (MSP)^25,26^ were conducted in samples from 1,469 men on the AutoDELFIA 1235 automatic immunoassay system (PerkinElmer, Turku, Finland) at the Wallenberg Research Laboratories, Department of Translational Medicine, Lund University, Skåne University Hospital, Sweden.^27,28^ All intra- and interassay coefficients of variation were < 9%.

### Statistical Analyses

Cox proportional hazards models were used to estimate the HRs and 95% CIs for prostate cancer incidence, and were used separately for death due to prostate cancer, using age as the underlying time variable. The slope of the Schoenfeld residuals over time was used to verify the proportionality of hazards.

All models were stratified by the particpant’s age at enrolment (< 50, 50-54, 55-59, 60-64, 65-69, and ≥ 70 years) and EPIC recruitment center. Multivariable models were adjusted for factors suspected to be associated with prostate cancer or vasectomy, including education (less than university, university graduate), smoking status (never, former, current), BMI (< 20, 20-24, 25-29, and ≥ 30 kg/m^2^), alcohol intake (< 8, 8-15, 16-39,and ≥ 40 g/d ethanol), and physical activity (inactive, moderately inactive, moderately active, active).^29^ Missing values were assigned to separate categories for education (3.8%), smoking status (1.5%), BMI (0.6%), and physical activity (0.8%), and missing indicators were used in the statistical models. Additional analyses were conducted adjusting for marital status (single, married, divorced, or widower); however, this information was only available for Germany and the United Kingdom. Adjustment was also made for protein from dairy sources (fourths) because this was previously found to be associated with prostate cancer in the EPIC cohort.^30^

Subgroup analyses were conducted according to patient’s age at vasectomy (< 38 *v* ≥ 38 years), time since vasectomy (< 25 *v* ≥ 25 years), by median BMI and alcohol consumption, physical activity, marital status, educational attainment, and smoking status. Additional analyses were conducted by tumor subtypes: stage (localized *v* advanced) and tumor grade (low-intermediate *v* high). Tests for heterogeneity in the association between vasectomy and prostate cancer were likelihood ratio tests for subgroup analyses and competing risk methods^31^ for stage and grade analyses. Country-specific associations were estimated using the Cox regression models and tests for heterogeneity were by likelihood ratio tests.

For a subset of 6,771 men in the EPIC-Oxford subcohort, for whom data on PSA testing were available, we investigated the suggestion that any association between vasectomy and prostate cancer is influenced by differences in the use of PSA testing in men who have undergone vasectomy.^2^ We used multivariable logistic regression to estimate the association of vasectomy with having a PSA test, adjusted for BMI and age at completion of questionnaire.

The cross-sectional association of vasectomy status with naturally logarithm-transformed plasma analyte concentrations (MSP, PSA [total, free, intact, free-to- total], and hK2) was evaluated using analysis of variance to compare geometric means adjusted for age at recruitment, BMI, recruitment center, and laboratory batch.

## RESULTS

Overall, 84,743 men were followed up for a median of 15.4 years (range, 0-20 years), of whom 4,377 developed prostate cancer. The mean age at recruitment was 53 years, which ranged from 50 years in Spain to 56 years in Denmark. The mean age at diagnosis of prostate cancer was 68 years, with a range of 65 years in Germany to 71 years in the United Kingdom. The proportion of men with self-reported vasectomy was 15% (n = 12,712), which ranged from 4.1% (n = 863) in Germany to 20.5% (n = 4,640) in the United Kingdom. For the 97.9% (n = 12,455) of men who had undergone vasectomy and who also provided age at vasectomy, median age at vasectomy was 38 years.

Compared with men without a vasectomy, men with a vasectomy were, on average, older at recruitment (54 years *v* 52 years), had a higher education level (university graduate, 33% *v* 26%), and were less physically active (14% *v* 19%). Men with a vasectomy were more likely to be married (91% *v* 80%). Vasectomy status also varied significantly by smoking status and alcohol consumption, although the magnitude of the differences was small (Table 1). Additionally, an analysis in the EPIC-Oxford subcohort showed that men who had undergone a vasectomy were 54% more likely to have had a PSA test when compared with men without a vasectomy (odds ratio, 1.54; 95% CI, 1.35 to 1.76).

**Table 1.**
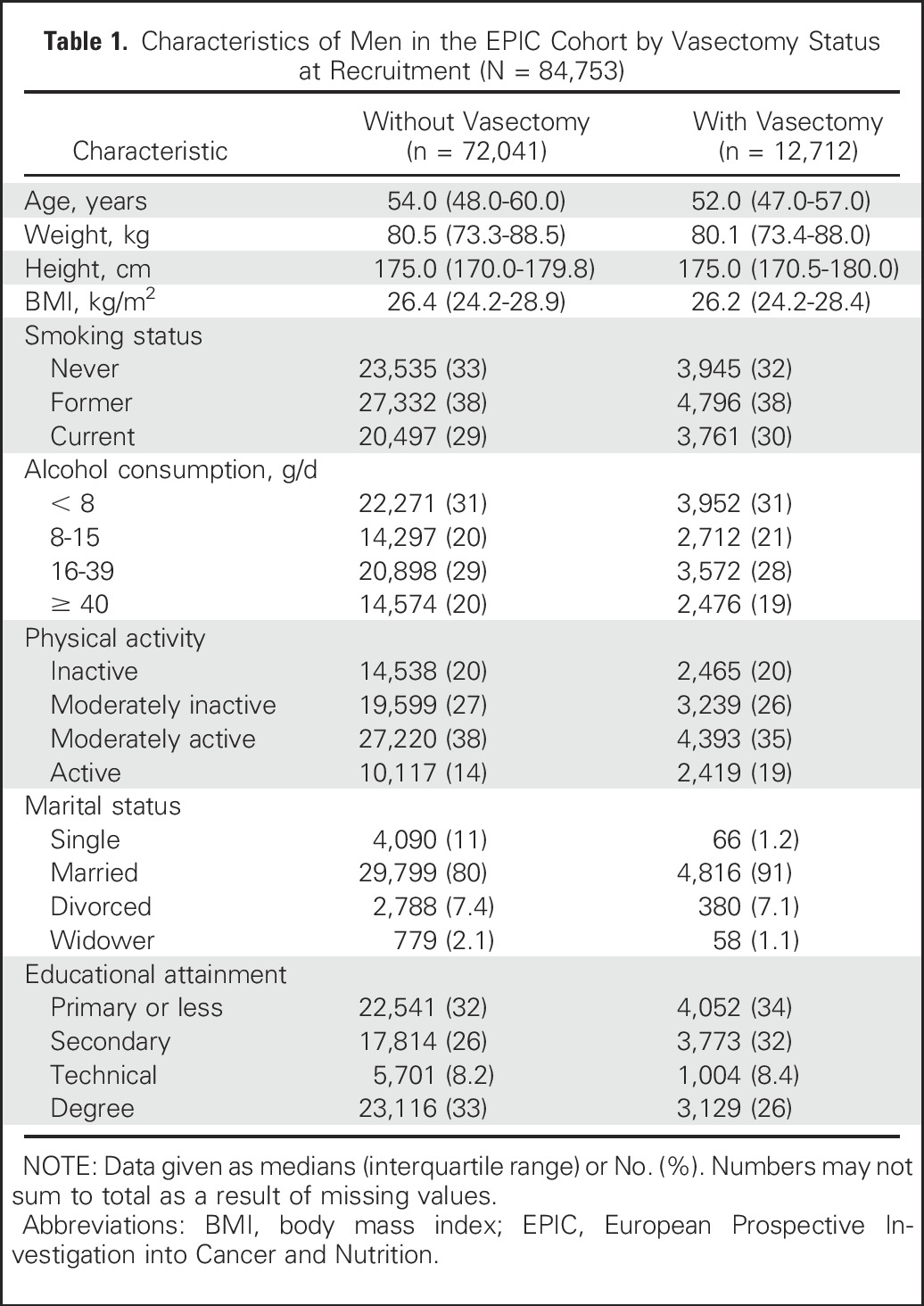
Characteristics of Men in the EPIC Cohort by Vasectomy Status at Recruitment (N = 84,753)

### Vasectomy and Prostate Cancer

Of the 4,377 men with prostate cancer for whom vasectomy status was available, 641 (14.6%) had a self-reported vasectomy at recruitment. Vasectomy was not significantly associated with prostate cancer risk after stratification by recruitment center and age at recruitment (HR, 1.05; 95% CI, 0.96 to 1.15). Additional adjustment for BMI, smoking status, marital status, educational attainment, alcohol consumption, physical activity, and protein from dairy sources did not alter results (HR, 1.05; 95% CI, 0.96 to 1.15). No evidence of heterogeneity was found in the association between vasectomy and prostate cancer by the stage of disease (*P* = .6) or recruitment country (*P* = .09; data not shown). However, there was evidence of heterogeneity by tumor grade (*P* = .02); vasectomy was associated with an increased risk of low-intermediate grade (HR, 1.14; 95% CI, 1.01 to 1.29) but not of high-grade prostate cancer (HR, 0.83; 95% CI, 0.64 to 1.07) (Table 2). Additionally, there was no significant association of vasectomy with death due to prostate cancer (HR, 0.88; 95% CI, 0.68 to 1.12).

**Table 2.**
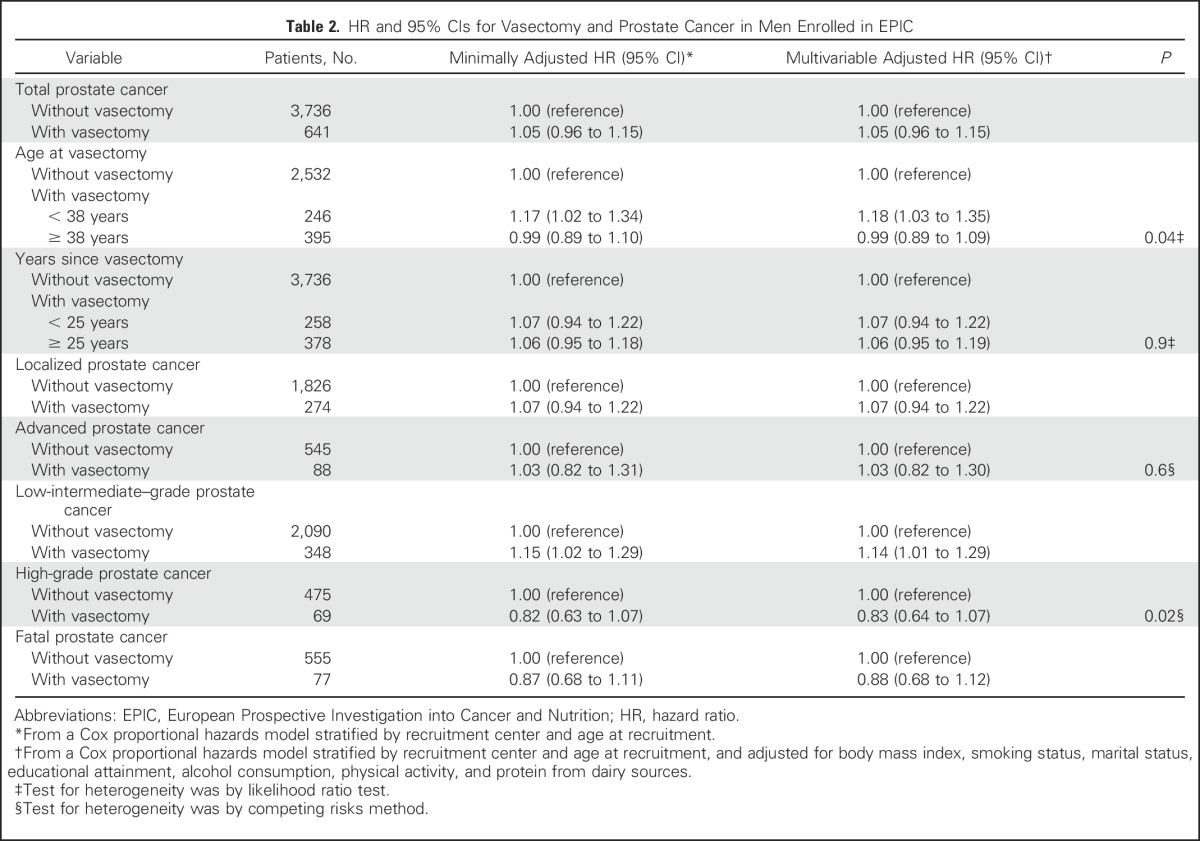
HR and 95% CIs for Vasectomy and Prostate Cancer in Men Enrolled in EPIC

There was significant heterogeneity in the association by median age at vasectomy (38 years) (*P* = .04). Compared with men who had not had a vasectomy, men who had a vasectomy when they were younger than the median age were at a significantly increased risk of prostate cancer (HR, 1.18; 95% CI, 1.03 to 1.35), whereas there was no significant association with prostate cancer in men who had a vasectomy when they were older than the median age (HR, 0.99; 95% CI, 0.89 to 1.09). There was also significant heterogeneity for the association of vasectomy with prostate cancer by median-defined strata of alcohol consumption (*P* = .03; data not shown). In men with below median alcohol consumption, those who had a vasectomy were at a significantly increased risk of prostate cancer (HR, 1.16; 95% CI, 1.02 to 1.31) compared with men without a vasectomy, whereas for men with above median alcohol consumption, vasectomy was not associated with prostate cancer (HR, 0.96; 95% CI, 0.84 to 1.08). No heterogeneity was observed for subgroup analyses by BMI, physical activity, marital status, educational attainment, or smoking status (data not shown). There was no heterogeneity (*P* = .9) in the association with prostate cancer risk by time since vasectomy.

### Circulating Concentrations of Seminal Proteins and Vasectomy

Compared with men without a vasectomy, men with a vasectomy had significantly higher concentrations of MSP (multivariable-adjusted geometric mean, 14.2 ng/mL (95% CI, 12.9 to 15.6) versus 12.8 ng/mL (95% CI, 12.4 to 13.1; *P* = .03). No significant differences by vasectomy status were observed for PSA level (total, free, intact, free-to-total) or hK2 (all *P* > .05; Table 3).

**Table 3.**
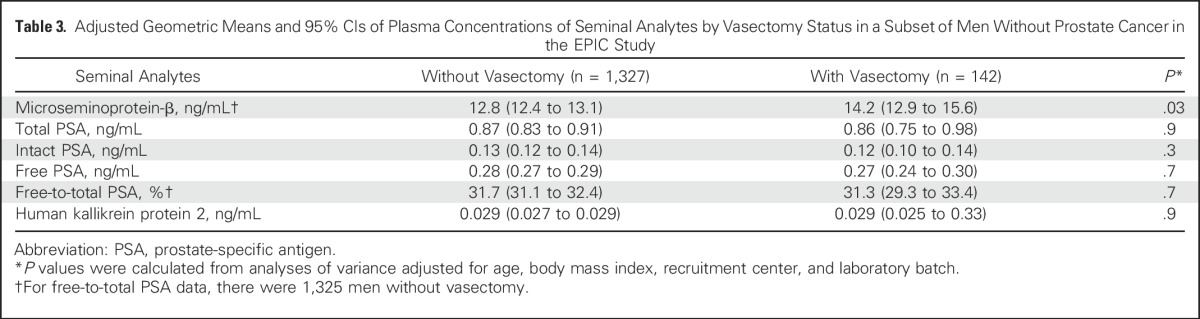
Adjusted Geometric Means and 95% CIs of Plasma Concentrations of Seminal Analytes by Vasectomy Status in a Subset of Men Without Prostate Cancer in the EPIC Study

## DISCUSSION

In this large, prospective European study, vasectomy was not associated with risk of prostate cancer overall, with risk for high-grade or advanced-stage tumors, or with death due to prostate cancer. However, there was some evidence that vasectomy may be associated with an elevated risk of low-intermediate–grade disease, and that having had a vasectomy is associated with also having had a PSA test.

Three of the seven previous cohort studies on vasectomy have reported an increased risk of prostate cancer in men with vasectomies.^4,10,32^ However, aside from the recent HPFS^4^ and the CPS-II cohort,^3^ all cohort studies have had a low number of incident cases with vasectomy (< 150), and so risk estimates have been subject to substantial uncertainty. The results from the HPFS cohort suggested a modest 10% elevated risk of overall prostate cancer and an elevated risk for aggressive tumor subtypes, with a 22% increased risk of high-grade and a 20% increased risk of advanced-stage tumors for men with vasectomies, compared with men without vasectomies. In contrast, both the current study and CPS-II^3^ found no significant association of vasectomy with prostate cancer overall, high-grade or advanced-stage disease, or death from prostate cancer. Thus, our findings do not support the previously hypothesized role of vasectomy as a risk factor for prostate cancer overall or for more aggressive tumors.

There is no established biologic rationale for an association of vasectomy with prostate cancer.^33^ During a vasectomy, the vas deferens is cut, blocked, or sealed to prevent the sperm from reaching the seminal fluid. Although previous studies have investigated a series of theoretical mechanisms that include immunologic response,^5^ changes to cell proliferation,^6^ and endocrine function,^4,7^ the biologic significance of these pathways in humans is unclear. This study addressed a recent suggestion that there may be differential regulation of seminal analytes in men after vasectomy^4,34^; among 1,469 men without prostate cancer, we found little evidence that vasectomy is associated with different blood plasma concentrations of PSA variants or hK2. However, significantly higher blood plasma concentrations of MSP were found in men with vasectomy compared with those without. MSP is a protein abundant in the seminal fluid,^35^ which has been found at significantly higher concentrations in the seminal plasma in infertile men when compared with fertile men.^36^ However, circulating concentrations have been previously inversely associated with prostate cancer^28^ and so our observation of higher circulating concentrations of MSP in men who had undergone vasectomy does not provide evidence in favor of vasectomy as a risk factor of prostate cancer.

A significantly increased risk of low-intermediate grade prostate cancer in men who had had a vasectomy in the current study might be at least partly explained by differences in the use of PSA testing. Men who receive a vasectomy may be more likely to attend health-care services and have their PSA level tested, and thus also be more likely to be diagnosed with prostate cancer, especially low-grade disease. Evidence for this hypothesis comes both from our finding that, in the EPIC-Oxford subcohort, men with a vasectomy were more likely to have had a PSA test than men without a vasectomy, and from the HPFS cohort, which also found that men with a vasectomy were more likely to have a history of PSA testing than men without a vasectomy.^4^ There was also some evidence for heterogeneity by age at vasectomy and alcohol intake, but the implications of these subgroup analyses are unclear.

Evidence from the European Randomized Study of Screening for Prostate Cancer suggests that when PSA testing is offered to all men, it reduces prostate cancer mortality by approximately 28% at 13 years of follow-up.^37^ If we assume that the use of PSA testing is 20% among men who have not had a vasectomy and 40% among men who have (data from EPIC-Oxford), it is possible that increased screening in the latter could result in a 5.6% reduced risk of death due to prostate cancer. This suggests that although it is possible that an adverse effect of vasectomy on the risk of potentially lethal prostate cancer is being partly masked by a beneficial effect of increased PSA testing, any such bias is likely small. Nevertheless, it remains a limitation of the current investigation that, because of limited information on PSA testing, we were unable to more fully address the role of PSA testing in the proposed association of vasectomy with prostate cancer. This should be considered in future studies.

Due to the lack of updated data collection for vasectomy status, it is possible that our results were biased by misclassification of men who had undergone vasectomy as being nonvasectomized. However, for the EPIC-Oxford subcohort, updated data on vasectomy status were available and showed that 5.1% of men without a vasectomy at baseline reported having had a vasectomy during the 10 years after recruitment. Furthermore, a recent report suggested that a small misclassification of men who had undergone vasectomy as nonvasectomized would likely result in only a minimal underestimate of any association^3^ of vasectomy with prostate cancer risk.

In conclusion, this investigation of 84,753 men in the EPIC cohort did not find a significant association between vasectomy and overall prostate cancer, high-grade or advanced-stage tumors, or death due to prostate cancer. The small increase in the risk of low-intermediate–grade prostate cancer in men who had had a vasectomy may be due to differences in health-monitoring behaviors.
